# Explainable and reliable kidney CT image classification using self-supervised DeiT-Tiny transformer

**DOI:** 10.3389/fmed.2026.1893419

**Published:** 2026-08-05

**Authors:** Sai Sri Hemantha Konala, Srinivas Koppu

**Affiliations:** School of Computer Science Engineering and Information Systems, Vellore Institute of Technology, Vellore, Tamil Nadu, India

**Keywords:** DeiT-Tiny transformer, DINO, Grad-CAM++, kidney classification, self-supervised learning

## Abstract

**Introduction:**

Kidney-related disorders are one of the global health concerns that require timely detection to prevent severe health complications. The use of computed tomography (CT) images for accurate classification of kidney diseases is important. However, it is challenging to differentiate between classes due to the subtle visual differences. This study introduces a novel two-stage deep learning architecture that integrates self-supervised representation learning with supervised classification for kidney CT image analysis using a publicly available kidney CT image dataset.

**Methods:**

In the first stage, the DINO framework with a Data-efficient Image Transformer (DeiT-Tiny) backbone is used to learn useful features from kidney CT images independent of labels. In the second stage, the pre-trained model is fine-tuned using labeled data to classify kidney abnormalities. To ensure model transparency and clinical trustworthiness, two explainable AI techniques are applied. Grad-CAM++ is used to highlight important regions contributing to predictions in kidney CT images. In addition, DINO’s inherent multi-head self-attention mechanism is analyzed across all attention heads to capture diverse attention patterns.

**Results and discussion:**

Experimental findings indicate that the proposed framework achieves strong classification performance, with a test accuracy of 99.16%, AUC-ROC of 99.99%, F1 score of 98.97%, precision of 98.90%, and recall of 99.05%, while also providing clear interpretability for automated kidney disease classification. External validation on a CT dataset from Iraq has yielded 97.03% accuracy, supporting the generalizability of the proposed framework.

## Graphical abstract

**Figure:**
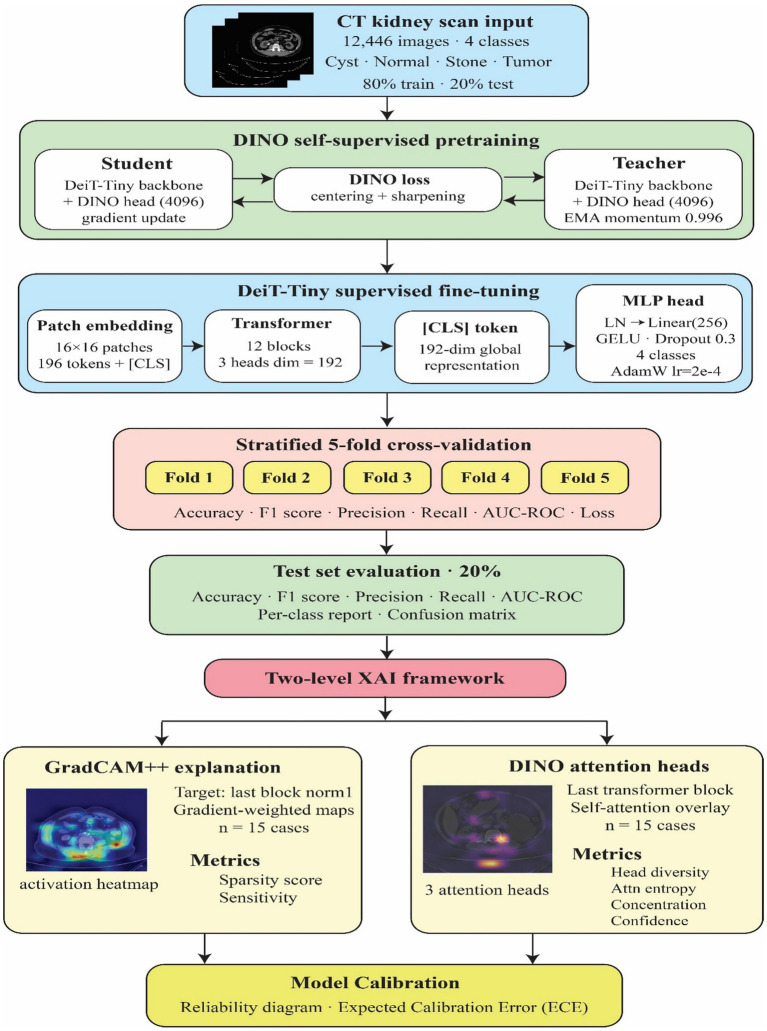
The proposed work.

## 1 Introduction

Renal disease, also referred to as kidney disease or nephropathy, involves pathological processes that gradually damage the kidneys and reduce renal performance (1). It is a global health concern, ranking as the 16th leading cause of mortality and affecting approximately 8–16% of the world’s population (2). Chronic kidney disease (CKD) is a prolonged disease involving progressive kidney damage with decreased filtration capacity of estimated glomerular filtration rate (eGFR) below 60 mL/min/1.73 m^2^ that leads to renal function impairment and requires either dialysis or transplantation for survival (3). Kidney disorders, including cysts, stones, and tumors, constitute a serious health concern. Renal cysts are fluid-containing lesions that disrupt normal kidney functioning; kidney stones result from mineral crystallization in urine, leading to severe discomfort and urinary tract complications; and tumors originate from renal tissues that result in various medical complications. Timely diagnosis and appropriate treatment are important in protecting kidney health (4). With consistent screening and timely medical treatment, kidney disease progression can be effectively managed or reversed in its early stages (5). Due to the shortage of nephrologists, it becomes difficult to deliver timely and effective medical treatment, even for symptomatic patients (6). Delayed diagnosis can lead to several complications, including kidney failure, making early detection essential. Medical imaging technologies, particularly computed tomography (CT) and magnetic resonance imaging (MRI), support doctors in the accurate identification of pathological abnormalities (7). Deep learning (DL) has significantly improved image processing through automated feature extraction and has resulted in enhancing the accuracy of essential tasks including segmentation, object detection, and image classification (8). Although DL has attained a high level of accuracy, it relies on large volumes of labeled data, which are often time-consuming and sometimes difficult to obtain. Even when labeled data are available, manual annotation can be biased and may not fully capture the diversity of important features. Self-supervised learning (SSL) handles this issue by learning diverse features from unlabeled data, which reduces bias and improves generalization (9, 10).

The major contributions of this study are the following:

- Developed a DINO-based self-supervised pre-training approach that utilizes a lightweight DeiT-Tiny Vision Transformer backbone to learn semantically meaningful feature representations from kidney CT scans, reducing reliance on manual medical annotations.
- Transferred the self-supervised representations to a supervised classification task using a custom MLP head.
- To support clinical trust and decision-making, the proposed model integrates a two-level XAI framework combining Grad-CAM++ explanation maps and DINO attention head analysis, quantitatively validated through Sparsity, Sensitivity, Entropy, Confidence, and Spatial Concentration metrics.
- Robust validation using 5-fold cross-validation was performed to evaluate generalization and the effectiveness of self-supervised pretraining.
- Confidence intervals, reliability diagrams, and calibration errors were computed to assess the reliability and calibration of the model predictions.
- The proposed framework achieved 97.03% accuracy on an independent CT dataset from Iraq, demonstrating its potential to generalize across different institutions and patient populations.

The article is organized into the following sections. Section 2 summarizes research in the field of deep learning and self-supervised learning methods for medical image classification. Section 3 outlines the proposed methodology, including the dataset, models, and evaluation methods. Section 4 reports the results and discussion of the proposed framework. Section 5 concludes the paper by summarizing the key outcomes. Section 6 examines the limitations of the current study and proposes areas for future research.

## 2 Literature survey

Pimpalkar et al. (11) have proposed a DL-based classification technique for a kidney CT dataset composed of 12,446 images. Using transfer learning models such as ResNet50, VGG16, AlexNet, and InceptionV3, the kidney images were categorized into Normal, Cyst, Tumor, and Stone classes. The method integrates watershed segmentation and Otsu’s binarization for precise boundary extraction and object separation, and the relief technique was incorporated for optimized feature selection. The Inception V3 model attained the highest classification accuracy of 99.96%. Obaid et al. (12) classified kidney images into cysts, stones, tumors, and normal, collected from multiple sources containing 27,151 images (urogram and CT scan) based on a DL approach. The framework was evaluated on 16 CNN models, including InceptionV3, ResNet variants, GoogleNet, VGG16, DenseNet, and DarkNet. Based on the fuzzy decision through the open score method, DarkNet53 was selected as the most efficient model with a classification accuracy of 99.69%. Anand et al. (13) have evaluated different ML techniques using different batch sizes (16 and 32) and optimization methods, including Adam and SGD, for early detection. This was followed by the classification of kidney tumors for accurate medical diagnosis using the CT scan kidney dataset with different split ratios (80:20 and 75:25). SVM has attained a maximum accuracy of 98.5% with the Adam optimizer and with a batch size of 32. Tamuly et al. (14) proposed a DL-based framework for kidney disease classification using five CNN models trained on a publicly available dataset of 5,578 kidney images. MobileNetV2 achieved the best performance with 99% precision, recall, and F1-scores across all classes. Saliency mapping was used to improve interpretability by highlighting the important regions that influenced the model’s predictions. Sharma et al. (15) introduced a hybrid CNN model to classify kidney CT images (12,446). By using a feature fusion technique, they combined features from ResNet101 and a custom CNN. The hybrid CNN achieved 99.73% training and 100% test accuracy. Pande and Agarwal (16) proposed a DL approach for detecting different types of kidney abnormalities from medical images using the YOLOv8n-cls model for class classification. The framework combined a feature extraction network with an object categorization detection head to improve abnormality recognition. The proposed model achieved an accuracy of 82.52%, specificity of 93.12%, precision of 85.76%, and F1-score of 75.52%. Islam et al. (17) developed an AI-based approach for kidney detection on a CT scan kidney dataset using CNN models including VGG16, Inception V3, and ResNet50, and also transformer models including Swin Transformer, CCT, and EANet. Experimental results showed that Swin Transformer outperformed other models with an accuracy of 99.3%. Meanwhile, VGG16 provided better explainability in identifying anatomical abnormalities. A comprehensive details of literature survey is provided in Table 1.

**Table 1:** Comparative analysis of existing research studies.

Authors	Dataset	Methodology	Challenges
Pimpalkar et al. (11)	CT kidney image dataset (12,446 images)	Watershed segmentation and Otsu’s binarization. Models used: VGG16, ResNet50, CNNAlexNet, and InceptionV3	The segmentation approaches can be limited by noise and low-contrast characteristics in CT images.
Obaid et al. (12)	27,145 kidneys sourced from five repositories (MRI and CT scans)	16 DL models, including ResNet, DenseNet, Inception, DarkNet, and MobileNet variants	The approach does not support multi-modal imaging data.
Anand et al. (13)	CT kidney image dataset considered only tumor (5077) and normal (2283).	SVM, KNN, DT, RF, and XGBoost.	The study lacked external validation and explainable AI support, limiting its interpretability and reliability.
Tamuly et al. (14)	5,578 kidney images from a publicly available dataset	Xception, ResNet152, MobileNetV2, NASNetMobile, and InceptionV3	The study relied on a single public dataset, which may limit the model’s generalization to real-world clinical data.
Sharma et al. (15)	CT kidney image dataset (12,446 images)	Hybrid CNN	Longer training time is required due to the complex hybrid architecture.
Pande et al. (16)	CT kidney image dataset (12,446 images)	YOLOv8n-cls	The proposed model limits performance comparison with other approaches.
Islam et al. (17)	CT kidney image dataset (12,446 images)	VGG16, ResNet50, InceptionV3, EANet, CCT, and Swin Transformer	The study uses Grad-CAM heatmaps for visual explainability, but quantitative interpretability analysis was not included.

Although DL-based image classification approaches have achieved impressive results, often reporting accuracies above 99%, there are still a few limitations that need to be addressed.

- Existing deep learning approaches rely heavily on fully supervised learning, requiring large annotated datasets that are challenging and time-intensive to acquire.
- High accuracies greater than 99% may not reflect real-world generalization due to dataset bias.
- Relying only on labeled data may limit the learning of robust and generalizable feature representations.

### 2.1 Self-supervised learning

SSL is a representation learning technique that allows models to learn generalizable and transferable feature representations directly from data without relying on explicit label supervision. Instead of using manual annotations, SSL defines learning objectives based on intrinsic relationships within the data by maintaining consistency between different augmented views of the same sample to capture meaningful structural and semantic properties of data. An important property of SSL is its flexibility regarding data annotations. Even when labeled data is available, SSL can be effectively used as a pre-training step with labels reserved for successive supervised training. As a result, the model learns invariant and generalizable representations independent of class-specific labels. The pre-trained model is further fine-tuned under supervised learning using labeled data to improve generalization and faster convergence (10).

Early contrastive self-supervised approaches such as SimCLR (18) and MoCo (19) learn visual representations by contrasting positive and negative sample pairs but are computationally expensive. Another limitation of these models is the false negative issue, where semantically similar samples are considered as negatives, resulting in lowering the effectiveness of representation learning. DINO (self-distillation with no labels) (20) is an SSL framework that introduces a student–teacher-based architecture to enforce representation consistency among augmented views of the same input. This method enables self-supervised pre-training without reliance on labeled data or contrastive negative samples.

Özbay et al. (21) developed an SSL framework (SSLSD-KTD) for kidney tumor classification using CT images from KAUH-Kidney (840 tumor images) and CT-kidney datasets (12,446 images). The proposed method combines a masked autoencoder (MAE) with self-distillation and, thereby, improves feature extraction through transferring global information between the decoder and encoder. The approach achieved 98.04 and 82.14%, which further improved to 99.82 and 95.24% with transfer learning. Kwasigroch et al. (22) has improved skin lesion classification using SSL on the ISIC2017 dataset with 2000 training images. The methodology incorporates multiple training strategies, including training from scratch, transfer learning, and SSL based on PIRL (pretext-invariant representation learning) through contrastive pretext tasks such as rotation and jigsaw. The obtained results showed that the performance of the model improved by averaging the outputs of the fully augmented networks and attained an accuracy of 86.16% and an ROC AUC of 0.856.

Tan et al. (23) introduced SSD-COVID, a self-supervised DL model for COVID-19 diagnosis on SARS-COV-CT and COVID-CT datasets using the masked autoencoder framework. To improve feature learning, the model uses image reconstruction and self-distillation loss during pre-training. The proposed framework achieved 97.78% accuracy on the SARS-COV-CT dataset and 81.76% on COVID-CT data; with external knowledge, accuracy improved to 99.6 and 95.27%. Wang et al. (24) proposed an SSL framework for eyelid malignant melanoma detection using PatchCamelyon (PCam) and ZJU-2 datasets. The proposed model employed the Bootstrap Your Own Latent (BYOL) framework with a ResNet50 backbone for optimal feature representation. Patch-level classification was performed using labeled and unlabeled data, with a random forest classifier and probabilistic heatmaps for interpretability. The proposed model attains a maximum accuracy of 98.1% at the patch level on the ZJU-2 dataset. Baasith et al. (25) developed an SSL-based vision transformer for lung disease classification using the Chexpert-v1.0-small dataset of 223,414 X-ray images. The ViT encoder is pre-trained using SSL and then fine-tuned in a supervised manner on 12 thoracic disease labels. Experimental findings demonstrate that SSL-ViT achieves an AUC of 0.75 and a micro-average recall of 0.73, indicating high sensitivity in detecting abnormal cases.

## 3 Methodology

This section presents the proposed methodology, which integrates self-supervised learning with the DeiT-Tiny transformer model as a backbone for kidney CT image classification. The framework includes dataset preparation, feature learning using SSL, model training, and explainability analysis to ensure reliable and interpretable predictions.

### 3.1 Dataset description and preparation

The dataset used in this research is the CT-kidney dataset (Normal, Cyst, Stone, and Tumor), which is a publicly accessible benchmark dataset from the Kaggle repository, comprising abdomen CT scan image data (12,446 images) categorized into four classes: Cyst, Normal, Stone, and Tumor. These are high-resolution JPEG image files stored in directories corresponding to each image class, with an additional CSV file containing metadata.^1^ The class distribution of the CT-Kidney dataset used in this study is represented in Table 2.

**Table 2:** Dataset class distribution.

Class	Number of images
Cyst	3,709
Normal	5,077
Stone	1,377
Tumor	2,283

The dataset was split into training and testing sets using an 80:20 ratio, with 80% used for training the model and 20% used to evaluate its performance. All four classes have enough samples for reliable training and evaluation. This split (9,956 training images, 2,490 test images) used stratified sampling (random_state = 42) to preserve class proportions. The test set was excluded from supervised training, hyperparameter tuning, model selection, and ablation studies. Five-fold stratified cross-validation was run on the training subset only (9,956 images) to check performance stability across partitions. The test set was not part of any CV fold and was used only for the final evaluation of the trained model. Augmentation was applied during DINO self-supervised training, with additional augmentation applied during supervised fine-tuning, to ensure evaluation reflects the model’s true capability. Images were resized to 224 × 224 pixels and normalized according to the ImageNet normalization parameters to fit the pretrained vision transformer input.

### 3.2 Proposed methodology

**Figure 1:**
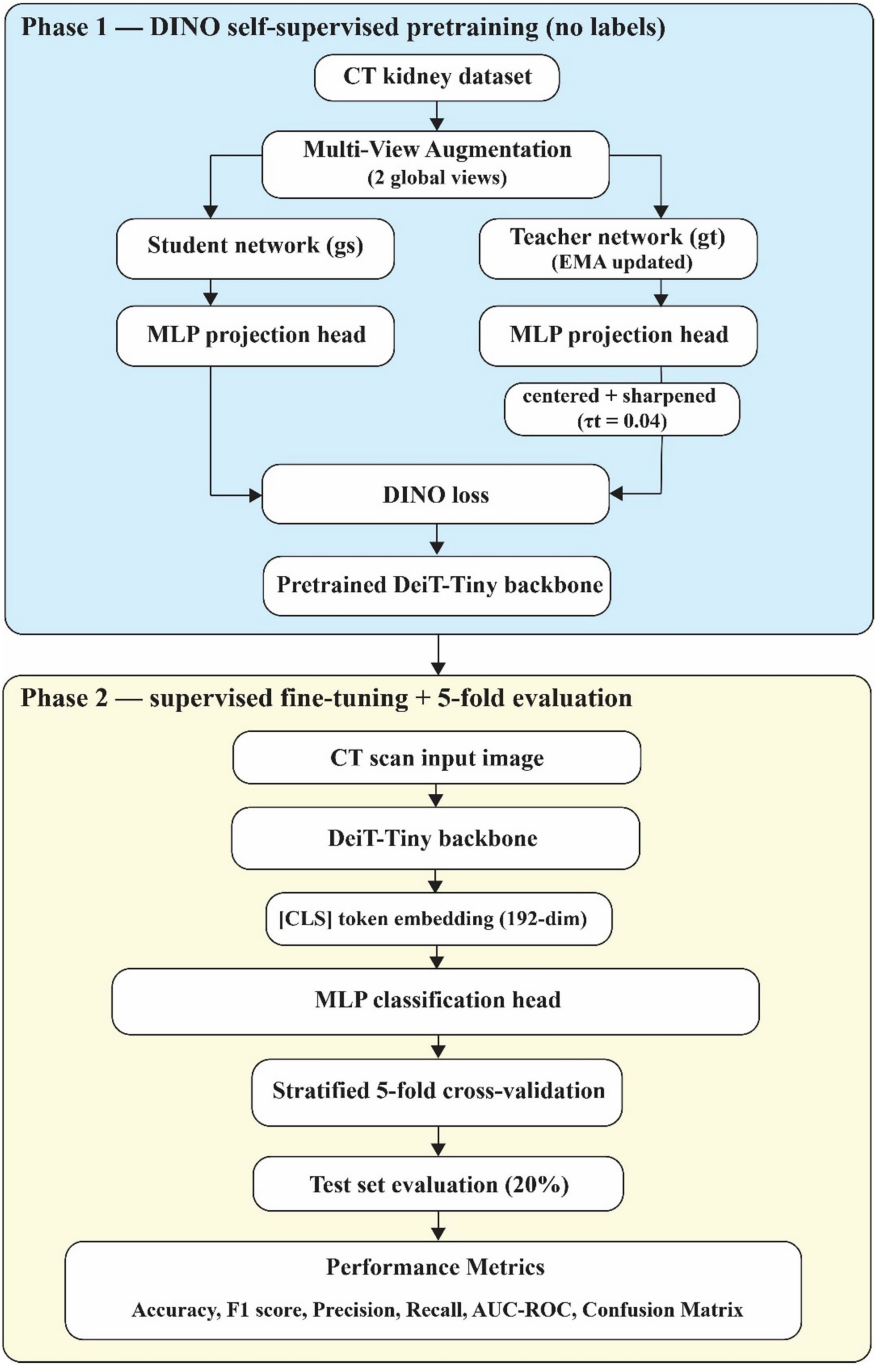
Overview of the proposed methodology for kidney CT image classification.

This section outlines a comprehensive description of the proposed two-stage framework for classification of kidney abnormalities from CT imaging data. The framework is structured in two sequential phases:

- Self-supervised representation learning through the DINO (Self-Distillation with NO labels) paradigm, and
- Supervised fine-tuning is performed using a multi-layer perceptron (MLP) classification head, evaluated under stratified k-fold cross-validation. The overall pipeline is illustrated in Figure 1.

### 3.3 Phase 1: Self-supervised pretraining using DINO

The first phase of the proposed framework involves training a Vision Transformer (ViT) backbone using Data-efficient Image Transformer (DeiT-Tiny) in a self-supervised manner using the DINO framework, as proposed by Caron et al. DINO is a self-distillation approach without labels by training a student model to align with the probability distribution of a momentum-updated teacher model using cross-entropy loss across multiple augmented views of the same image. The student network is optimized through backpropagation, while the teacher network is updated using an exponential moving average (EMA) of the student network parameters. Although labeled data is available, the model is pretrained without labels, enabling it to learn strong representations from the data itself.

#### 3.3.1 Backbone architecture: DeiT-Tiny

The backbone model used is the DeiT-Tiny model, with the patch size set to 16 and input resolution set to 224 × 224, using the Timm library. The DeiT-Tiny model generates a 192-dimensional embedding from the [CLS] token of the final transformer layer. The student and teacher models share this identical model architecture. The model is trained from scratch, with random initialization, without any pre-training on the ImageNet data, to ensure that all representational capacity is learned from the data distribution of the CT kidney images during the self-supervised pretraining phase. The teacher model is set to be identical to the student model at the beginning of the training process using the state dictionary, to ensure that the exponential moving average (EMA) is started from the same state (26).

#### 3.3.2 Dual-view augmentation

As per the DINO approach proposed by Mathilde Caron et al., the training images are augmented independently to generate two different views of the image: x_1_and x_2,_ from the same original image x, which are then passed through the student and teacher networks in a cross- prediction manner. The student is trained to predict the teacher’s output for the complementary view, and vice versa. The augmentation process applied to each view includes resized cropping (global crops at scale 0.4–1.0), random horizontal flipping, color jitter, greyscale conversion, Gaussian blur, and optional solarization, in accordance with the original DINO augmentation technique. This multi-view approach is important as the network learns invariances by forcing the network to output consistent representations of the images despite the structural differences between the views of the images.

#### 3.3.3 DINO projection head

To enable self-distillation, a shared projection head is appended to every backbone, projecting the 192-dimensional [CLS] token embedding into a higher-dimensional feature space. The projection head is designed as follows:

- A two-layer MLP with a hidden dimension of 2048, followed by a bottleneck layer producing an intermediate representation of dimension 512. GELU activations are applied between both linear layers.
- L_2_ normalization of the 512-dimensional vector before the final linear projection ensures representations lie on the unit hypersphere to prevent representation collapse.
- A weight-normalized layer projects the normalized vector of dimension 512 to the final dimension of 4,096 for better stability during training.

The DINO projection head maps the backbone embedding h to a high-dimensional representation z. The final DINO representation is computed using Equation 1 as proposed by Caron et al. (20):


$$z=W*\frac{f\left(h\right)}{{\left\|f\left(h\right)\right\|}_{2}}$$ (1)

where:

- *h* ∈ ℝ^192^ — [CLS] token embedding from the DeiT-Tiny backbone
-
f(h) = *W*₂ · GELU(*W*₁h) ∈ ℝ^512^ — two-layer MLP bottleneck (*W*₁ ∈ ℝ^2048^ˣ^192^, *W*₂ ∈ ℝ^512^ˣ^2048^)
-
$\frac{f\left(h\right)}{{\left\|f\left(h\right)\right\|}_{2}}\;$ = L2 normalization onto unit hypersphere
- *W* ∈ ℝ^4096^ˣ^512^ is weight-normalized linear projection
- z ∈ ℝ^4096^ represents final DINO representation

#### 3.3.4 DINO loss with centering

The DINO training objective uses a cross-entropy loss between a sharpened probability distribution generated by the teacher model and the probability distribution estimated by the student model in the projected representation space, as shown in Equation 2 (20)


$$L_{DINO}=-\sum P_{t}\left(y_{2}\right)\log P_{s}\left(y_{1}\right)-\sum P_{t}\left(y_{1}\right)\log P_{s}\left(y_{2}\right)$$ (2)

where:

- *P_t_* and *P_s_* denote the probability distribution of the teacher and student networks, respectively, which are computed using Equations 3, 4
- *Y_1_* and *Y_2_* are two augmented views of the same input image,

$$p_{t=}\;softmax\left(\frac{z_{t-C}}{T_{t}}\right)$$ (3)

$$p_{s}=softmax\left(\frac{z_{s}}{T_{s}}\right)$$ (4)

z *_t_* and z*_s_* ∈ ℝ^4096^ are the raw logits from the teacher and student projection heads, respectively, and c ∈ ℝ^4096^ is a centering vector maintained as an exponential moving average of teacher outputs to avoid collapse. The loss is computed symmetrically across both views, with the teacher distribution sharpened using a low temperature *T_t_ =* 0.04 and the student distribution sharpened using a low temperature, *T_s_ =* 0.1.

#### 3.3.5 Exponential moving average teacher update

The teacher network is not trained using backpropagation. After every gradient step, the model parameters are updated using an EMA of the student model weights represented in Equation 5 (20):


$$\theta_{t}\leftarrow \lambda \theta_{t}+\left(1-\lambda \right)\theta_{s}$$ (5)

where:

-
$\theta_{t}\;and$ $\theta_{s}\;$ represents the weights of the teacher and student networks, respectively, through EMA
- *λ* = 0.996 is the EMA momentum coefficient.

The center vector is updated using an EMA to stabilize the teacher outputs in DINO, as shown in Equation 6 (20).


$$c\leftarrow m.c+\left(1-m\right).mean\left(Z_{t1},Z_{t2}\right)$$ (6)

where *m* is the momentum coefficient (0.9), and $Z_{t1},Z_{t2}\;$ are the teacher network outputs from different augmented views.

#### 3.3.6 Optimization and training configuration

The student network is optimized using the AdamW optimizer (learning rate 2 × 10^−4^, weight decay 1 × 10^−4^), with PyTorch automatic mixed precision (AMP) to enhance computational efficiency. Gradient norms are clipped to 3.0 to prevent exploding gradients, and pre-training is conducted for 25 epochs. After training, student backbone weights are extracted and saved, excluding the projection head.

Table 3 summarizes hyperparameter configurations for DINO self-supervised pretraining, including the optimizer, learning rate, batch size, temperature values, and EMA momentum, which were kept consistent across all evaluated architectures.

**Table 3:** Hyperparameter configuration for DINO self-supervised pretraining.

Hyperparameter	Value
Backbone	DeiT-Tiny (patch16, 224 × 224)
Embedding dimension	192
Projection head output dim	4,096
Student temperature (τₛ)	0.1
Teacher temperature (τₜ)	0.04
Center momentum	0.9
EMA momentum (λ)	0.996
Optimizer	AdamW
Learning rate	2 × 10^−4^
Weight decay	1 × 10^−4^
Gradient clip norm	3.0
Pretraining epochs	25
Mixed precision	FP16 (AMP)

Algorithm 1 outlines the DINO self-supervised pretraining process used to learn feature representations from unlabeled kidney CT images.

Algorithm 1: DINO self- supervised pretrainingInput: Unlabeled CT kidney image dataset D = {*y*_1_,*y*_2_,.*y*_N_}.Output: Pretrained backbone weights $\theta_{s.}$ 1. Initialize student network *g_s_* with parameters $\theta_{s}$ 2. Initialize teacher network *g_t_* with parameters $\theta_{t}$ 3. Freeze teacher parameters $\theta_{t}$ 4. Initialize center vector *c* ⟵ 05. for epoch = 1 to *E* do6. for each batch x ⊂ *D* do7. Generate augmented views $y_{1},y_{2}$ from *y*8. *Z_s1_ = g_s_*(*y_1_*), *Z_s2_ = g_s_*(*y_2_*) $9.p_{s}\left(y_{1}\right)=softmax\left(\frac{z_{s1}}{T_{s}}\right)$ , $\;p_{s}\left(y_{2}\right)=softmax\left(\frac{z_{s2}}{T_{s}}\right)$ 10. with no_grad ():11. *Z_t1_ = g_t_*(*y_1_*), *Z_t2_ = g_t_*(*y_2_*)12. $p_{t}\left(y_{1}\right)=softmax\left(\frac{z_{t1}-c}{T_{t}}\right)$ , $\;\;p_{t}\left(y_{2}\right)=softmax\left(\frac{z_{t2}-c}{T_{t}}\right)$ 13. Compute DINO loss:14. ${ℒ}_{DINO}=-\sum P_{t}\left(y_{2}\right)\log P_{s}\left(y_{1}\right)-\sum P_{t}\left(y_{1}\right)\log P_{s}\left(y_{2}\right)$ 15. Update $\theta_{s}$ using backpropagation: $\theta_{s}\leftarrow \theta_{s}-\eta ▽L$ 16. Update $\theta_{t}$ using EMA: $\theta_{t}\leftarrow \lambda \theta_{t}+\left(1-\lambda \right)\theta_{s}$ 17. Update center: *c⟵ m.c +* (1-*m*). *mean* (*Z_t1,_ Z_t2_*), *m =* 0.918. end for19. end for20. Save pre-trained backbone weights $\theta_{s}\to$ DINO_kidney_backbone.pth

### 3.4 Phase 2: Supervised fine-tuning with MLP classification head

After completing self-supervised pretraining, the student backbone is employed as a feature extractor, with a multi-layer perceptron (MLP) classification head attached in place of the original linear classifier.

#### 3.4.1 MLP classification head

The classification head replaces the single linear layer with a deeper MLP, as it captures non-linear feature interactions, allowing more effective discrimination of complex patterns in medical images, while a linear layer is limited to simple linear decision boundaries. The MLP effectively models complex patterns from the 192-dimensional feature vector and classifies it into four categories. It includes a LayerNorm applied to the input, followed by a fully connected layer (192 → 256), GELU activation, dropout (rate = 0.3) for regularization, and a final layer (256 → 4). During training, the model outputs are directly utilized with CrossEntropyLoss, which internally applies a softmax function to obtain class probabilities.

#### 3.4.2 Training configuration

The classification head is trained using the AdamW optimizer with a learning rate of 2 × 10^−4^ and a weight decay of 1 × 10^–4,^ together with the cross- entropy loss. The entire model (backbone with MLP head) is fine- tuned for 25 epochs, with no layers frozen. The complete fine-tuning configuration is summarized in Table 4.

**Table 4:** Hyperparameter configuration for supervised fine-tuning (DeiT-Tiny).

Hyperparameter	Value
Dropout rate	0.3
Batch size	16
Optimizer	AdamW
Learning rate	2 × 10^−4^
Weight decay	1 × 10^−4^
Scheduler	None
Epochs	25
Backbone initialization	DINO-pretrained weights
Backbone frozen during fine-tuning	NO (fully trainable)

### 3.5 Evaluation strategy

A stratified 5-fold cross-validation is performed to check how well the model generalizes to unseen data. The dataset is divided into five folds, with each fold maintaining a similar class distribution as the entire dataset, so the evaluation is fair and balanced. For each fold, the model is reinitialized from the DINO-pre-trained backbone before training to ensure that learning from previous folds does not affect the current fold’s training, which in turn prevents data leakage across folds. After training for 10 epochs per fold, the model is tested on the held-out validation fold. Finally, results from all five rounds are aggregated so that every sample is used for testing exactly once, leading to a more accurate and reliable measure of the model’s performance. After cross-validation, the model is then evaluated on the test set comprising 20% of the original dataset to provide an unbiased assessment of its generalization ability on unseen data, reporting accuracy, precision, recall, F1 score, AUC_ROC, and per-class confusion matrix.

Algorithm 2 summarizes the supervised fine-tuning process used to train and evaluate the DINO-pretrained model.

Algorithm 2: Supervised fine-tuning using 5-fold cross-validation and test set evaluationInput: Input: Labeled CT kidney dataset D = {(x_i_, yi)}, y_i_ *ϵ* {0,1,2,3}, Hold-out test set T (20%)Pretrained backbone weights $\theta_{s}$ (from Algorithm 1)Output: Performance metrics (Accuracy, Precision, Recall, F1, AUC, Confusion matrix)1. Partition D into K = 5 stratified folds: {F_1_, F_2_, F_3_, F_4_, F_5_}2. Initialize collectors: all_labels = [], all_preds = [], all_probs = []3. for fold k = 1 to 5 do4. Load pretrained backbone $\theta_{s}\;$ from dino_kidney_backbone.pth5. Attach randomly initialized MLP head:LayerNorm →FC (192 → 256) → GELU →Dropout (0.3) → FC (256 → 4)6. Set training set Tr = D\F_k_; validation set Val = F_k_7. for epoch = 1 to 10 do8. for each batch (x, y) ϵ Tr do9. h = backbone (x)10. logits = MLP_head (h)11. L = CrossEntropyLoss (logits, y)12. Update $\theta_{s}$ through backpropagation13. end for14. end for15. for each batch (x, y) ϵ Val do16. probs = softmax (model(x))17. preds = argmax (probs)18. Append y → all_labels, preds → all_preds, probs → all_probs19. end for20. end for21. Compute CV performance metrics (Accuracy, Precision, Recall, F1, AUC,Confusion matrix).22. Evaluate model on test set T:23. probs = softmax (model(x)); preds = argmax(probs)24. Compute: Accuracy, Precision, Recall, F1, AUC, Confusion matrix.

### 3.6 Baseline models for comparative evaluation

A set of advanced deep learning models is selected for comparison, and each model is discussed in detail in the following sections.

ViT-B/16 is a Vision Transformer (27) that divides an image into small 16 × 16 patches and considers each patch like a token, similar to words in a sentence. These patches are then processed through transformation layers that use self-attention, allowing the model to understand relationships among different image components. Unlike CNNs, which mainly focus on local regions, Vision Transformers (ViT) capture information from the entire image at once. This makes them useful for medical imaging tasks, where important patterns may appear across different regions (28). The model is built on the standard attention mechanism proposed by Ashish Vaswani et al. (29) is expressed in Equation 7:


$$Attention\left(Q,\;K,\;V\right)=softmax\left(\frac{QK^{S}}{\sqrt{d_{k}}}\right)V$$ (7)

where:

*Q, K,* and *V* represent query, key, and value matrices, facilitates the modelling of long-range dependencies across image patches.

$QK^{S}$ calculates the similarity between the query and all keys using the dot product.

$d_{k}$ is the dimension of key vectors for normalizing the similarity scores.

V is the actual feature information of the input.

Swin-Tiny is a hierarchical Vision Transformer that partitions images into small patches (4×4) and performs self-attention within local windows (7 × 7). It uses a shifted window mechanism that helps the model effectively capture both local and global features. Its multi-stage structure also progressively reduces the spatial size while increasing the depth of features, similar to CNNs (30) The CaiT-XXS24-224 model, introduced by Touvron et al., is an extension of Vision Transformers that improves their depth and stability by using Class-Attention (CA) layers and LayerScale. Unlike Vision Transformers, CaiT separates the processing of image patches from the class token to learn more meaningful and discriminative features. This is particularly useful for CT scan datasets, where distinguishing minor variations among cysts, tumors, stones, and normal tissues is challenging. Its lightweight configuration captures important global relationships within the image and also helps prevent overfitting (31).

LeViT-128 s model is a lightweight vision transformer architecture that integrates the efficiency of CNN with the representational capacity of self-attention, making it suitable for medical imaging tasks such as CT scan analysis. Its hybrid design helps in working faster with less computational cost, while still capturing both local texture patterns and global contextual relationships for identifying different features in kidney CT scans. Because of this balance between speed and accuracy, this model is a good choice for real-time or resource-constrained deployment (32).

### 3.7 Explainability framework or model interpretability techniques

Deep learning models are often challenged because it is hard to clearly understand how they make decisions, which is commonly referred to as the black box problem (33). Although these models yield good performance metrics, it is hard to understand how they arrive at their decisions (34). In clinical CT imaging, model interpretability is crucial for diagnostic reliability and for earning the trust of radiologists. The application of two Explainable AI (XAI) techniques: GRAD-CAM++ (Gradient-Weighted Class Activation Mapping++) for spatial explanation of predictions and DINO attention visualization for explaining self-supervised representations, provides interpretability at both prediction-level and representation-level.

#### 3.7.1 Grad-CAM++

It is an extension of Grad-CAM (35) that identifies the regions that are most responsible for prediction by measuring how the class score changes with respect to the underlying feature maps, and combines these gradient signals into a spatial saliency map (36). We evaluate the quality of these explanations using two quantitative metrics: sparsity and sensitivity.

Sparsity quantifies how focused the saliency map is on a specific region rather than spreading across the total image, as shown in Equation 8 (37):


$$Sparsity=1-\frac{\left|\left\{x:L^{c}\left(x\right)>a\right\}\right|}{H\times W}$$ (8)

where *x* denotes a pixel, *a =* 0.5 is the activation threshold, and *H × W* is the total number of pixels. *L* is the heatmap image produced, and *c* is the predicted class. A higher sparsity score indicates that the model pinpoints a precise, localized lesion region, which is important in CT imaging where pathological regions reside in only a small portion of the scan.

Sensitivity measures how stable the explanation remains when minor noise is added to the input image, as shown in Equation 9 (38):


$$Sensitivity=E_{\delta ∼N\left(0,\sigma^{2}\right)}Z$$ (9)

$$Z=\left[{\left\|L^{c}\left(y+\delta \right)-L^{c}\left(y\right)\right\|}_{1}\right]$$ (10)

where *δ* represents Gaussian noise with standard deviation *σ* = 0.05, and Z, as defined in Equation 10, denotes the mean absolute difference between the original and perturbed explanation maps. A lower sensitivity score indicates that the model’s explanations are robust and consistent.

#### 3.7.2 DINO attention head analysis

Vision Transformers pretrained with DINO (20) are able to learn meaningful and interpretable attention maps on their own, without any labeled data. Each attention head in the last transformer block focuses on different parts or patterns in the input image, including object boundaries, semantic regions, textures, or global contextual relationships, and computes how much the CLS token represents the global image summary and also attends to every other token in the sequence. For each head h, the attention distribution is computed using Equation 11:


$$a^{i}=softmax\left(\frac{q_{CLS}^{i}.{\left(K^{i}\right)}^{T}}{\sqrt{d_{k}}}\right)\in {\mathbb{R}}^{N}$$ (11)

where *i* represents the attention head, $q_{CLS}^{i}$ is the CLS token query, $K^{i}$ denotes the key vectors of all tokens. Their dot product calculates similarity scores, which are scaled by $\sqrt{d_{k}}$ and then passed through a softmax to generate attention weights over all *N* tokens.

We quantify attention quality using four metrics:

Head Diversity: It measures how differently each head attends to the image, as expressed in Equation 12 (20)


$$Diversity=1-\frac{1}{T\left(T-1\right)}\underset{j\neq k}{\sum }p\left(a^{j},a^{k}\right)$$ (12)

where *T* is the number of heads, $p\left(a^{j},a^{k}\right)$ is the similarity between the attention maps of heads *j* and *k*

Attention Entropy: Measures the spread of the attention distribution, where lower values represent concentrated focus and higher values indicate more uniform attention, as defined in Equation 13 (39)

$Entropy^{k}=-\frac{1}{\log N}\overset{N}{\underset{j=1}{\sum }}{\hat{a}}_{j}^{k}\log$ ( ${\hat{a}}_{j}^{k}+\in$ ) (13)

where *N* is the total number of tokens, ${\hat{a}}_{j}^{k}$ is the normalized attention weight of the *j*-th token for head *k*, and ∈ is a small constant added to avoid numerical issues while computing the logarithm.

Attention Confidence: Quantifies how strongly an attention head prioritizes its most attended token, reflected by the maximum attention weight, as formulated in Equation 14 (39).


$$confidence^{k}=\max_{j}{\hat{a}}_{j}^{k}$$ (14)

The max operation selects the highest attention value, representing the most strongly attended token by that corresponding head *k*.

Spatial concentration: Quantifies the fraction of image patches required to accumulate 80% of the attention mass, representing how spatially focused the attention, as expressed in Equation 15 (20).


$$Concentration^{k}=\frac{\left|P_{0.8}^{k}\right|}{N}$$ (15)

where $P_{0.8}^{k}$ is the smallest set of patches with cumulative attention >80%, N is the total number of patches.

## 4 Results and discussion

The experiments were conducted using GPU-accelerated computation on dual NVIDIA Tesla T4 GPUs provided by the Kaggle platform. This section presents a comprehensive evaluation of the proposed framework, including the analysis of DINO loss convergence, comparative classification performance across different transformer-based architectures, and robustness assessment using 5-fold cross-validation. Explainability results are provided to interpret the model’s decision-making process.

### 4.1 Classification performance

This section presents the classification performance of the proposed DINO-pretrained DeiT-Tiny framework. First, the DINO training loss is analyzed to examine the convergence of the self-supervised pretraining process. The performance of the proposed model is then compared with several classification models using validation accuracy. Finally, test set results, five-fold cross-validation metrics, and confusion matrix analyses are presented to evaluate the accuracy, robustness, and consistency of the framework.

#### 4.1.1 DINO pretraining loss curve

**Figure 2:**
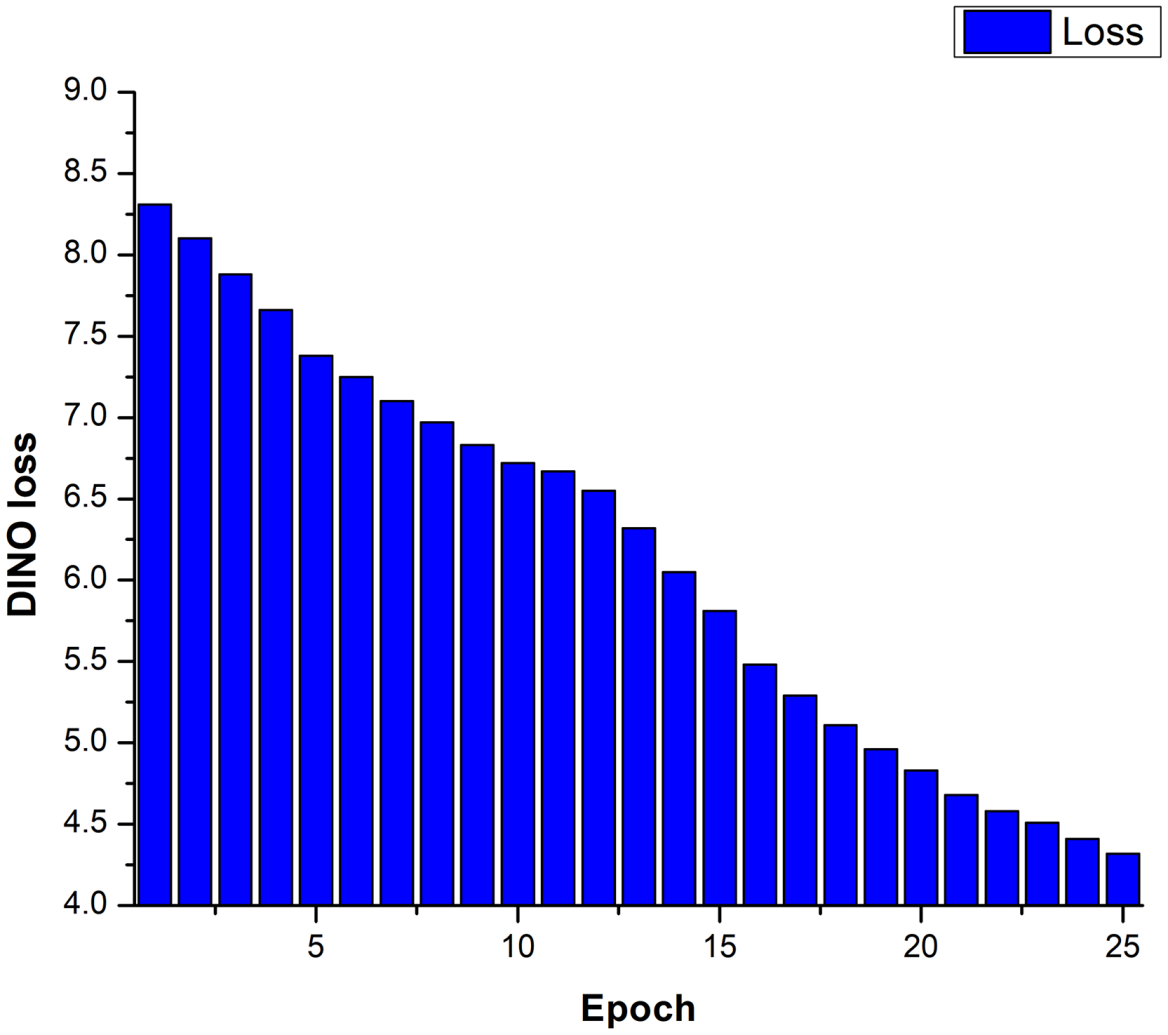
DINO loss across training epochs.

The effectiveness of the self-supervised pretraining stage was evaluated using the DINO training loss curve over 25 epochs, as illustrated in Figure 2.

Figure 2 demonstrates the progression of DINO loss across epochs. It is observed that a steady and linear decline in loss was observed starting from 8.31 (initial epoch) to 4.32 (final epoch, i.e., 25). This trend indicates that the model has displayed its ability to progressively learn meaningful representations. It is important to note that during the early phase, the loss has decreased rapidly, indicating the effective learning of global structures. As the training progressed, the rate of loss decrease slowed, suggesting feature refinement and convergence of the model.

#### 4.1.2 Validation accuracy comparison of five models

**Figure 3:**
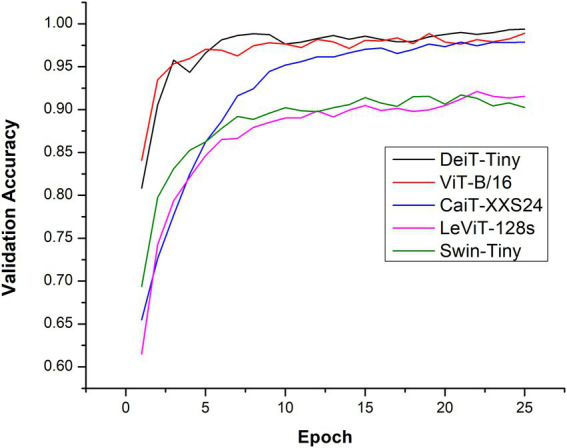
Classification accuracy of different transformer models.

To compare the effectiveness of different classification architectures, the validation accuracy of the five transformer models pretrained with DINO (DeiT-Tiny, ViT-B/16, CaiT-XXS24, LeViT-128 s, and Swin-Tiny) was compared to identify the model with the best classification performance on the kidney CT dataset represented in Figure 3.

Figure 3 presents the obtained validation accuracy of the evaluated five models over 25 epochs. All models have exhibited a clear upward trend in accuracy during the early stages of training. This reflects the effectiveness of DINO-pretrained representations in guiding the learning process. Of the five models, DeiT-Tiny showed strong and stable performance throughout training. Despite minor fluctuations between epochs, this lightweight transformer model achieved the highest accuracy of 99.38% at epoch 25, highlighting its effectiveness for kidney CT image classification. ViT-B/16 stood second and has demonstrated strong and consistent performance across epochs (starting from 5 epoch till 25). CaiT-XXS24 has shown a gradual improvement in accuracy and has attained an accuracy of 0.9787 at epoch 21. Swin-Tiny and LeViT-128 s have recorded the performance with a maximum accuracy of 0.9172 and 0.9212, respectively. Overall, DeiT-Tiny and ViT-B/16 are found to be efficient in achieving rapid convergence and high accuracy.

#### 4.1.3 Test set results

The proposed DeiT-Tiny model using DINO’s self-supervised framework was evaluated on a held-out test set comprising 2,490 samples. The model showed strong performance, achieving an accuracy of 99.16%. In terms of macro-averaged metrics, the F1 score was 98.97%, precision of 98.9%, recall of 99.05%, and AUC-ROC of 99.99%. At the per-class level. AUC-ROC values reached 99.99% for Cyst, Normal, and Stone, and 99.97% for Tumor, demonstrating the model’s strong discriminating ability over the classes. These results highlight the model’s generalization capability to unseen data with very few misclassifications across all four kidney conditions.

**Figure 4:**
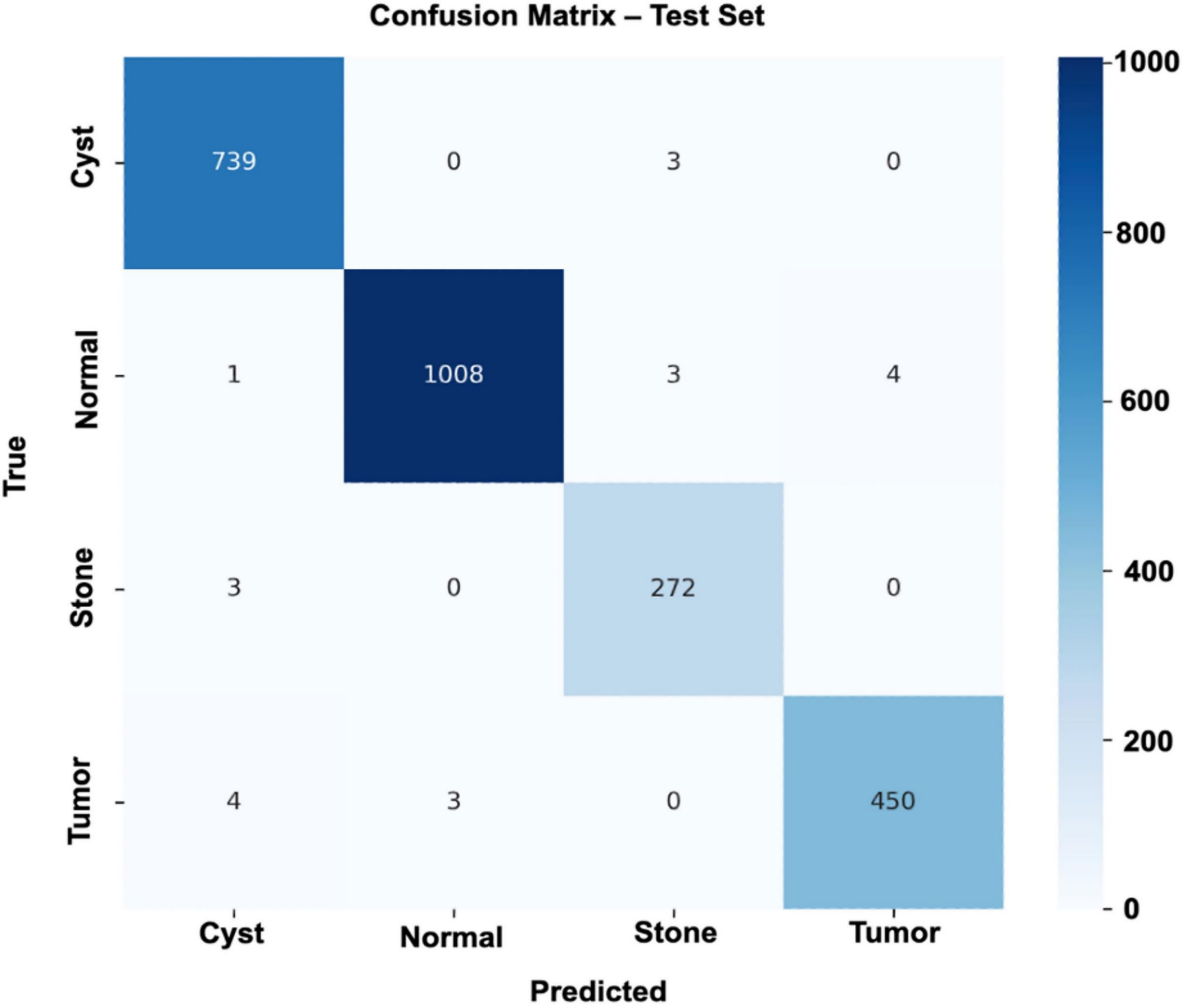
Confusion matrix for test set results.

Table 5 presents the per-class performance of the proposed model on the test dataset. The results exhibit good performance across all four kidney abnormality classes with very few misclassifications. The Confusion matrix obtained for the test set is given in Figure 4.

**Table 5:** Per-class classification performance on the test set.

Class	Precision	Recall	F1 score	AUC-ROC
Cyst	0.99	1.0	0.995	0.9999
Normal	1.0	0.99	0.995	0.9999
Stone	0.98	0.99	0.985	0.9999
Tumor	0.99	0.98	0.985	0.9997

The Confusion matrix shows that the proposed model accurately classified most of the test images across all four kidney conditions.

#### 4.1.4 Five-fold cross-validation results

Next, the proposed model’s consistency and generalization performance were evaluated. For this, a 5-fold cross-validation was performed. Moreover, fold-wise validation accuracy and validation loss were analyzed across different data splits.

**Figure 5:**
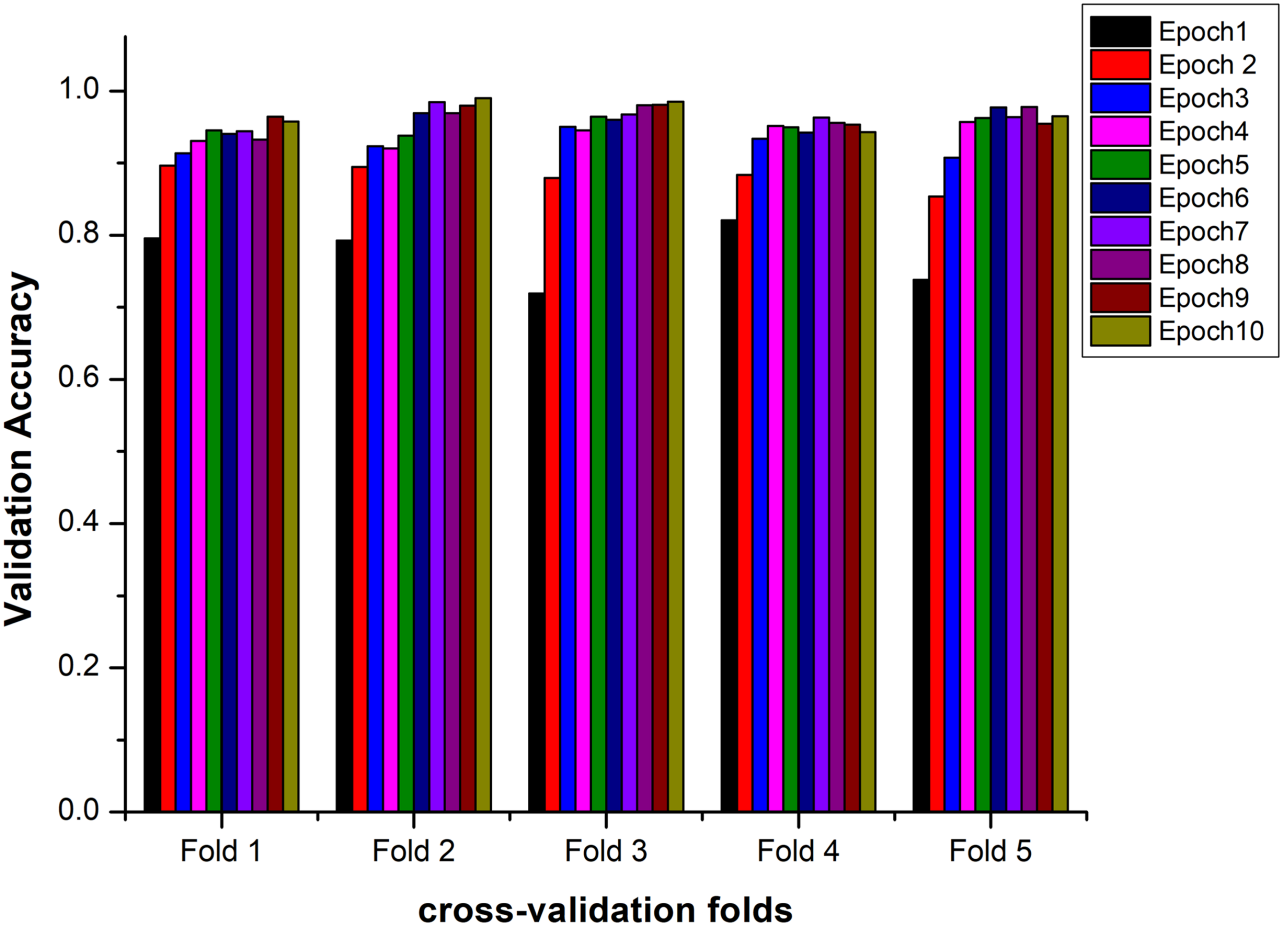
Fold-wise validation accuracy of the proposed model.

Figure 5 demonstrates the validation accuracy obtained across all folds. As can be seen, for all the folds, initially, the model achieved accuracies between 0.719 and 0.8, which have steadily increased between 0.9427 and 0.99 in later epochs. This analysis has revealed that the highest accuracy of 0.99 was achieved by Fold 2. The performance of other folds (Folds 1, 3, 4, and 5) was comparable, with final accuracies of 0.9573, 0.9849, 0.9427, and 0.9647, respectively. The low variability between different folds suggests reliable and consistent model performance that represents good generalization capability. Overall, the model has demonstrated stable convergence without any major instability, with some slight fluctuations in the later epochs.

**Figure 6:**
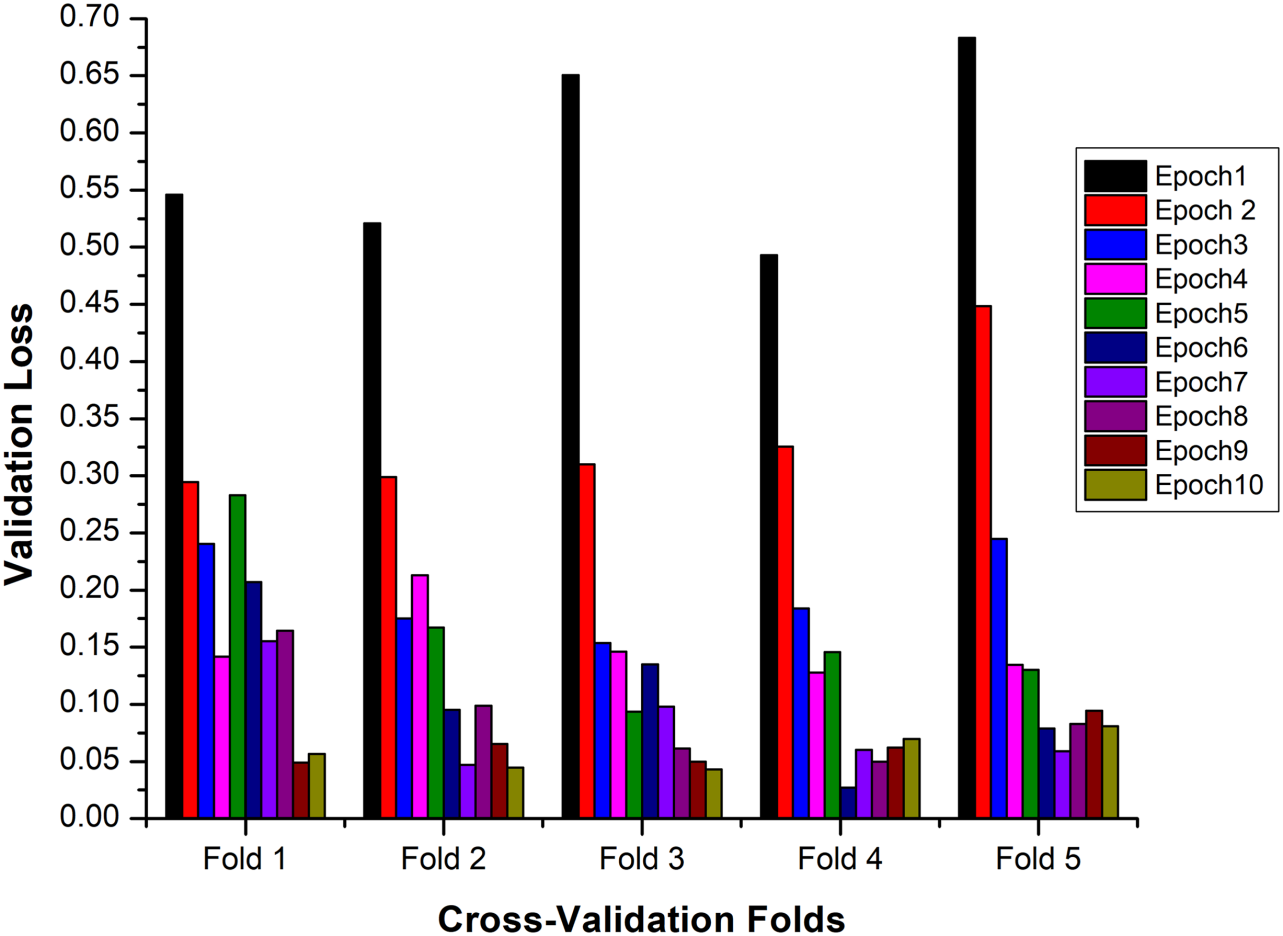
Fold-wise validation loss of the proposed model.

Figure 6 displays the validation loss across all folds, showing a clear decrease in the trend across the epochs. Epoch 1 had the highest loss values (0.5461, 0.5208, 0.6504, 0.493, and 0.6831) across all the Folds (Fold 1, Fold 2, Fold 3, Fold 4, and Fold 5). Importantly, with the progress in training, the loss has gradually reduced irrespective of the folds but at different epochs. Fold 3 and Fold 2 have shown a strong reduction by reaching 0.0431 and 0.0445 at epoch 10. The overall steady decrease in validation loss has shown that the model is learning well, with consistent performance across all the folds. The best validation performance, mean, and standard deviation achieved across the five folds during cross-validation are represented in Tables 6, 7.

**Table 6:** Best validation performance across five-fold cross-validation.

Fold	Epoch	Val_Acc	Val_F1 score	Val_Precision	Val_Recall	Val_AUC-ROC
1	9	0.9849	0.9748	0.9777	0.9823	0.9994
2	10	0.99	0.9858	0.9874	0.9843	0.9993
3	10	0.9849	0.981	0.9846	0.9776	0.9997
4	7	0.9829	0.9776	0.9813	0.9743	0.9992
5	8	0.9779	0.9701	0.9720	0.9686	0.9973

**Table 7:** Mean and standard deviation of validation performance over five-fold cross-validation.

Performance metrics	MEAN ± STD
Val_Acc	0.9841 ± 0.0043
Val_F1 score	0.9789 ± 0.0058
Val_Precision	0.9806 ± 0.0060
Val_Recall	0.9774 ± 0.0063
Val_AUC-ROC	0.999 ± 0.0010

**Figure 7:**
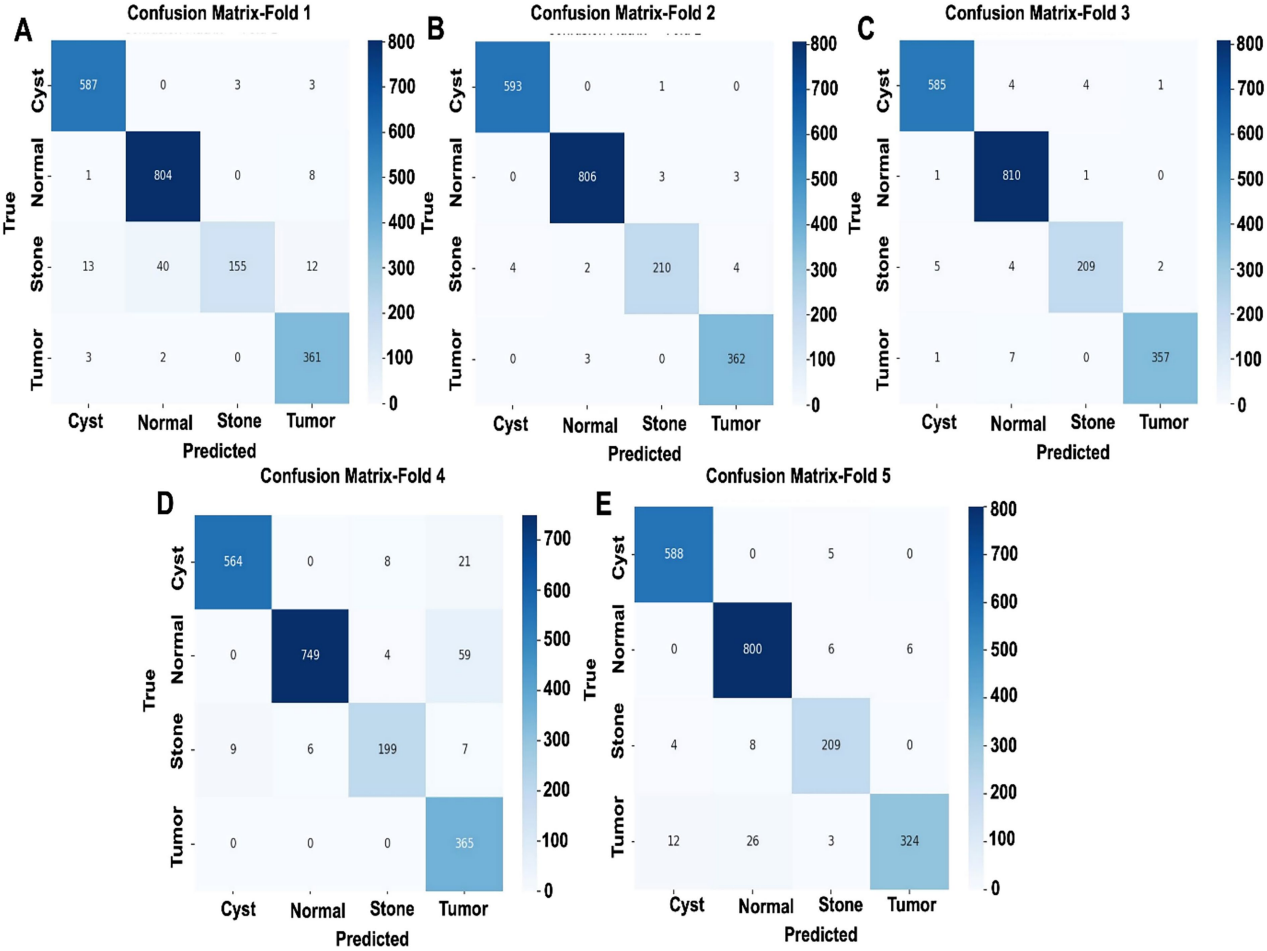
Confusion matrices for the five-fold cross-validation results: **(A)** Fold 1, **(B)** Fold 2, **(C)** Fold 3, **(D)** Fold 4, and **(E)** Fold 5.

The overall cross-validation results (Tables 6, 7, and Figure 7) show that the proposed model performed well across all five folds. The confusion matrices for five folds indicate that most kidney CT images were correctly classified with few errors only. The mean and standard deviation values indicate stable performance, whereas the best fold metrics highlight the model’s ability to accurately identify all four kidney disorders.

### 4.2 Computational complexity

Predictive accuracy is an important factor in model selection, but practical deployment also requires consideration of computational efficiency. To provide a more comprehensive evaluation, we compared the five architectures evaluated in this study, including DeiT-Tiny, ViT-Base, CaiT-XXS24, LeViT-128 s, and Swin-Tiny, all pretrained using DINO self-supervised learning, in terms of parameter count, multiply-accumulate operations (MACs), training time, per-image inference latency, and test accuracy, as represented in Table 8.

**Table 8:** Computational complexity of the evaluated architectures.

Model	Params (M)	MACs (M)	Inference (ms/img)	Total train time (min)	Accuracy (%)
DeiT-Tiny + DINO	5.58	34.33	6.93	89.42	99.16
ViT-Base +DINO	86.00	201.01	19.23	280.00	98.47
CaiT-XXS24 + DINO	11.79	40.53	14.96	215.34	97.47
LeViT-128 s + DINO	7.11	57.56	7.05	79.88	93.65
Swin-Tiny + DINO	27.72	62.23	11.52	164.45	90.84

As shown in Table 8, DeiT-Tiny achieved the highest accuracy (99.16%) while also requiring the fewest parameters (5.58 M) and MACs (34.33 M) among the five architectures. It additionally recorded the lowest inference latency (6.93 ms/image) and the second-shortest training time (89.42 min). In comparison, ViT-Base used substantially more parameters (86.00 M) and MACs (201.01 M) and achieved a slightly lower accuracy (98.47%) and required considerably longer training time. DeiT-Tiny provided the best balance between predictive performance and computational efficiency among the architectures evaluated.

### 4.3 Statistical significance analysis (McNemar’s test)

Performance metrics including accuracy, F1-score, and AUC-ROC summarize model performance, but they do not show whether differences between architectures are statistically meaningful or simply due to chance. To assess this, McNemar’s test (40) was applied, which compares two classifiers on paired predictions from the same test set.

McNemar’s test uses a 2 × 2 contingency table built from the disagreements between two models on the same test set. For each pair of models, four counts are recorded: cases both models classify correctly (n_11_), cases both misclassify (n_00_), cases the first model gets right but the second gets wrong (n_10_), and cases the second gets right but the first gets wrong (n_01_). The test depends on the discordant counts, n_10_ and n_01._ The continuity-corrected test statistic is given by Edwards (41), as shown in Equation 16:


$$\chi^{2}={\left(|n_{10}-n_{01}|-1\right)}^{2}/\left(n_{10}+n_{01}\right)\;$$ (16)

Here, $\chi^{2}$ follows a chi-square distribution with one degree of freedom under the null hypothesis of equal error rates between the two models. This test is particularly suitable for deep learning, where training multiple copies of each model to estimate performance variability is often computationally expensive.

McNemar’s test was applied between each pair of the five architectures (DeiT-Tiny, ViT-Base, CaiT-XXS24, LeViT-128 s, and Swin-Tiny). All models were evaluated on the same held-out test set (*n* = 2,490), satisfying the paired-sample assumption required by McNemar’s test.

Nine pairwise comparisons were statistically significant (*p* < 0.05), with several exhibiting extremely small *p*-values (*p* < 10^−25^). The only non-significant comparison was between DeiT-Tiny and ViT-Base (*p* = 0.0545). Despite ViT-Base having more parameters (86.0 M), its performance was not statistically different from that of DeiT-Tiny (5.6 M). Furthermore, DeiT-Tiny required fewer MACs (34.3 M vs. 201.0 M) and achieved lower inference latency (6.93 ms vs. 17.98 ms per image). These findings indicate that DeiT-Tiny provides comparable predictive performance with considerably lower computational cost.

The results, including the McNemar’s χ^2^ statistic, exact p-values, and discordant prediction counts for each pairwise comparison, are provided in Table 9.

**Table 9:** McNemar’s test for pairwise model comparison.

Model A	Model B	acc_A %	acc_B %	n10	n01	Statistic	P_value	Significant
DeiT-Tiny + DINO	Swin-Tiny + DINO	99.16	90.84	222	17	174.13	9.293× 10^−40^	Yes
ViT-Base + DINO	Swin-Tiny + DINO	98.47	90.84	216	26	147.61	5.7802× 10^−34^	Yes
DeiT-Tiny + DINO	LeViT-128 s + DINO	99.16	93.65	149	14	110.16	9.0415× 10^−26^	Yes
CaiT-XXS24 + DINO	Swin-Tiny + DINO	97.47	90.84	211	46	104.65	1.4546× 10^−24^	Yes
ViT-Base + DINO	LeViT-128 s + DINO	98.47	93.65	149	29	79.56	4.6871× 10^−19^	Yes
CaiT-XXS24 + DINO	LeViT-128 s + DINO	97.47	93.65	149	54	43.53	4.1813× 10^−11^	Yes
DeiT-Tiny + DINO	CaiT-XXS24 + DINO	99.16	97.47	54	14	22.37	2.2513× 10^−6^	Yes
LeViT-128 s + DINO	Swin-Tiny + DINO	93.65	90.84	179	109	16.53	4.7855× 10^−5^	Yes
ViT-Base + DINO	CaiT-XXS24 + DINO	98.47	97.47	61	36	5.94	0.0148	Yes
DeiT-Tiny + DINO	ViT-Base + DINO	99.16	98.47	34	19	3.70	0.0545	Yes

The McNemar’s test results indicate that the performance differences between the five architectures were statistically significant for most model pairs. The only exception was the comparison between DeiT-Tiny and ViT-Base. DeiT-Tiny achieved comparable performance with lower computational cost and provides a favorable balance between accuracy and efficiency.

### 4.4 Model calibration and reliability assessment

**Figure 8:**
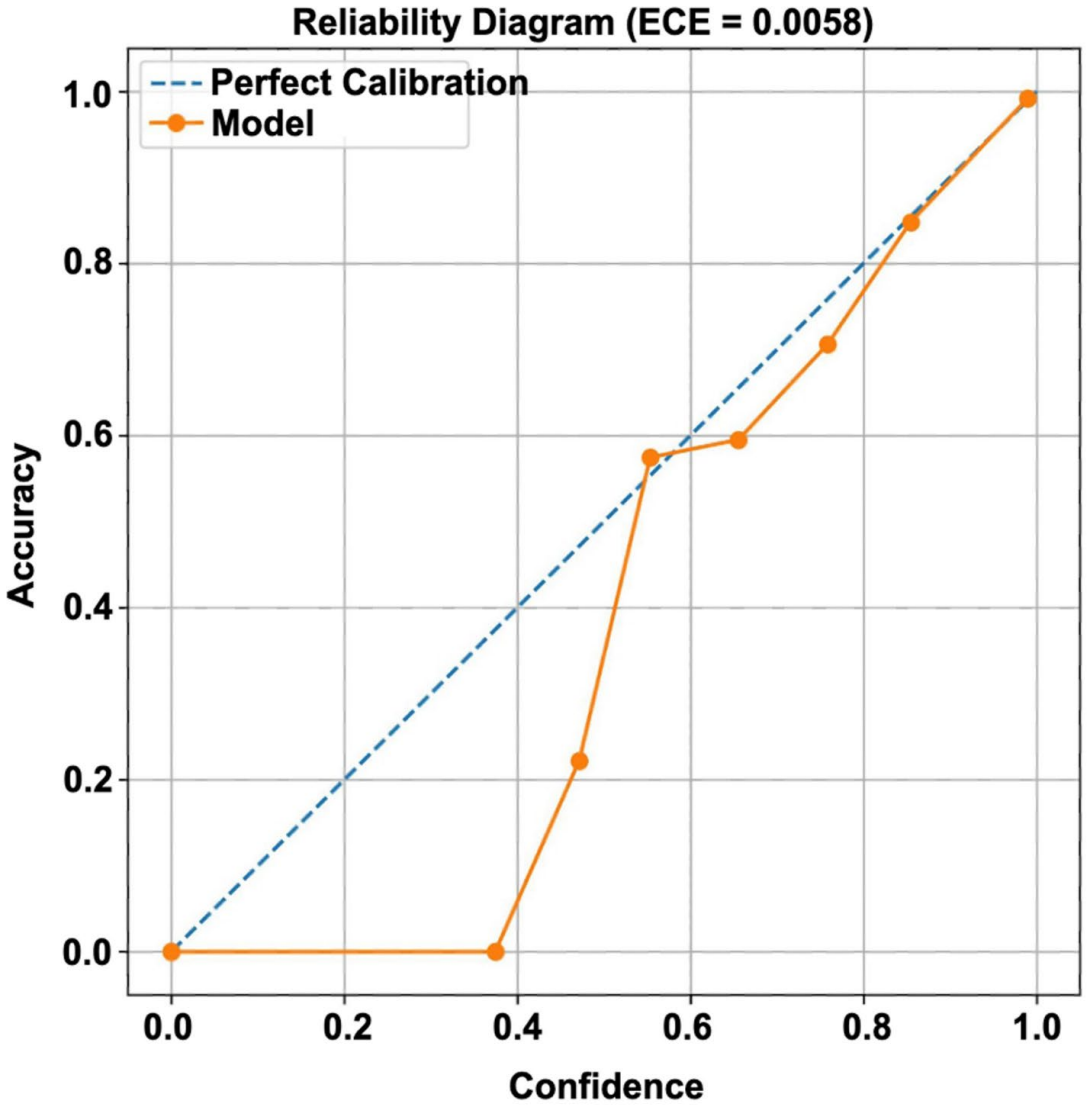
Reliability diagram of the proposed model.

To ensure reliable and consistent predictions, calibration and statistical validation were performed. Model calibration was assessed using a reliability diagram (Figure 8) and Expected Calibration Error (ECE), yielding a low value of 0.0058, which indicates that the predicted probabilities are well-calibrated and reliable. In addition, statistical validation was performed using the bootstrap-based confidence intervals. The best validation accuracy from each fold resulted in a mean accuracy of 0.9841, F1 score of 0.9738, precision of 0.9806, Recall of 0.9774, AUC-ROC of 99.90 with associated 95% confidence intervals (0.9803, 0.9875), (0.9738, 0.9832), (0.9757, 0.9851), (0.9725, 0.9822), (0.9981, 0.9995) confirming the consistency and robustness of the model’s generalization performance.

### 4.5 Explainability analysis

**Figure 9:**
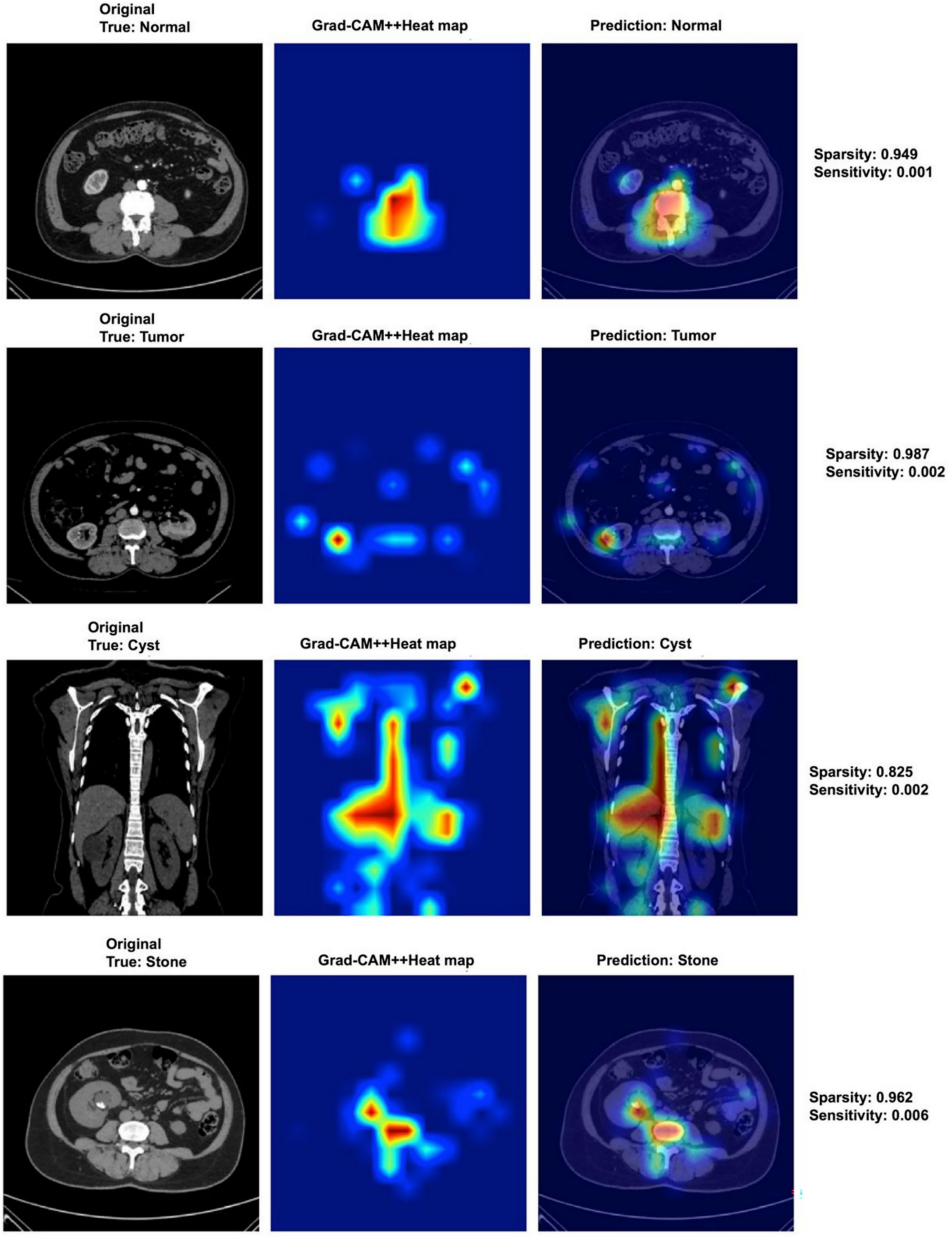
Grad-CAM++ heatmaps showing model attention for selected kidney CT samples.

The Grad-CAM++ was employed to provide visual explanations of the model’s predictions for the 15 representative samples. Figure 9 illustrates the regions that contribute significantly to the classification outcomes for a subset of images. Moreover, additional insights into the quality and reliability of the generated visual explanations were provided.

The mean sparsity value of 0.7820 indicates that the activation maps are well-localized, with the model focusing on a limited and relevant set of regions rather than diffusing attention over the entire image, showing the ability of the model to identify important regions. The low mean sparsity of 0.0218 shows that the highlighted regions remain stable under small input changes, indicating the robustness of the generated explanations. The visualizations provided by Grad-CAM++ visualizations demonstrate precision and consistency, enabling reliable interpretation in clinical contexts.

**Figure 10:**
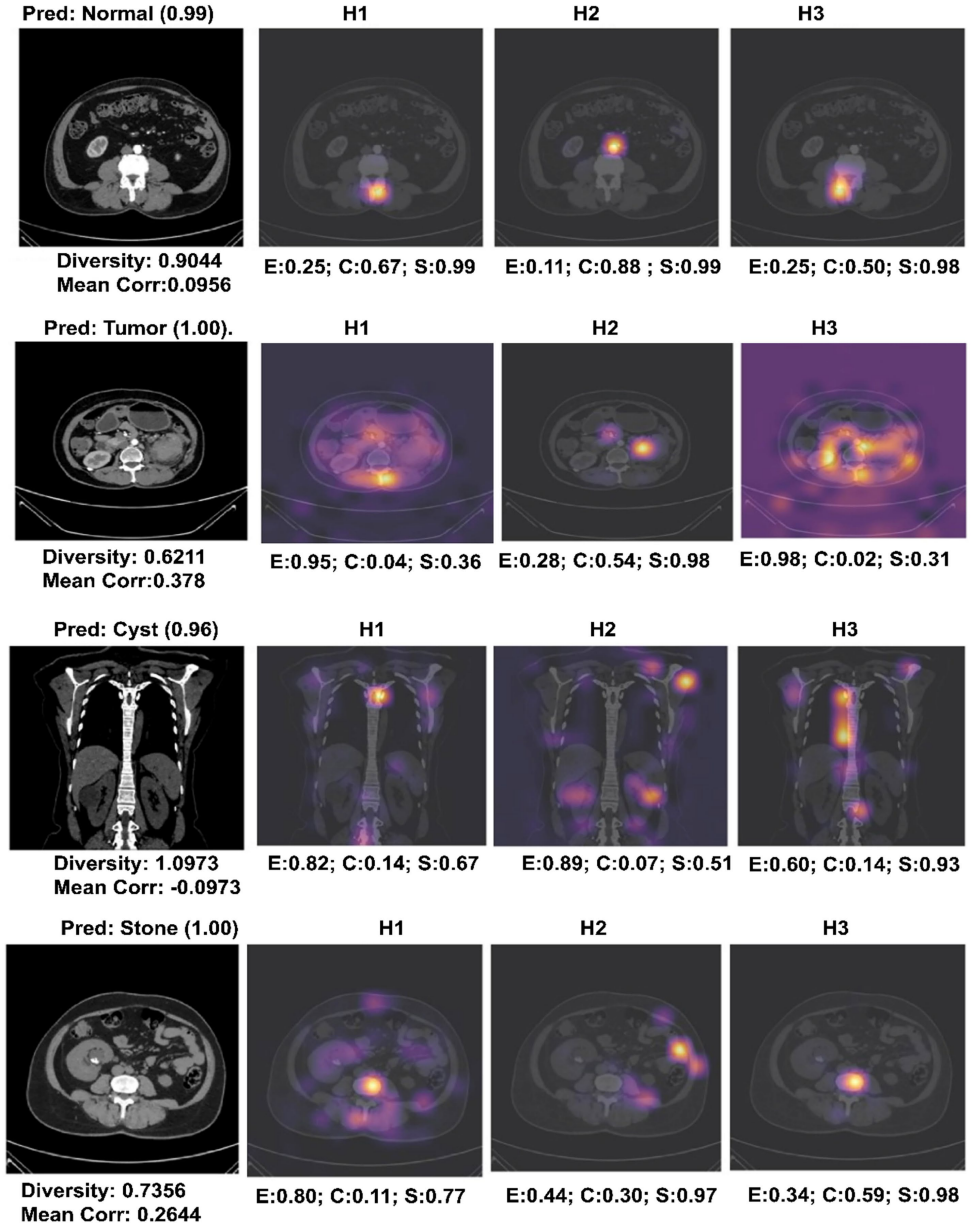
Multi-head attention maps showing model focus for different kidney abnormalities.

The attention-based analysis using DINO heads is employed to visualize how the model focuses on different regions of the input images. The evaluation is performed on 15 samples; a subset of representative examples is presented in Figure 10 to illustrate the attention patterns, giving a clear understanding of interpretability and readability of the learned representations.

The diversity mean (0.9317) represents that the attention heads have captured different and complementary features, hence reducing redundancy in the representations learned. The entropy mean (0.3332) suggests a balanced distribution of attention, where the model considers both important regions and surrounding context. The confidence mean (0.5661) shows that the attention is not concentrated on a single region, thereby reducing dependence on limited features. The concentration mean (0.8956) demonstrates that the model is able to focus attention on relevant areas of the image. These results suggest that the model achieves a good balance between diversity and focus, supporting meaningful and reliable interpretation of its predictions.

### 4.6 Ablation study

To assess the contribution of individual components within the proposed architecture, an ablation study was carried out by selectively activating the MLP and SSL Modules. The baseline model DeiT-Tiny ViT achieved a test accuracy of 92.45% without MLP and SSL. Adding the MLP alone improved performance to 94.33%, showing that it helps the model to learn better feature representations. Similarly, incorporating SSL without the MLP increased accuracy to 98.02%, showing that self-supervised learning helps the model learn more useful features. The final proposed model, combining both MLP and SSL, achieved the highest test accuracy of 99.16%, demonstrating that both components work together to improve overall model accuracy.

### 4.7 Generalizability assessment using an independent dataset

To assess the generalizability of the proposed methodology, an additional experiment was conducted using the Kurdistan Kidney Stone Dataset, an independent public dataset collected in Iraq. The dataset contains two classes, Stone and Non-stone, and was developed to support AI-based stone detection studies. The dataset can be accessed at: https://www.kaggle.com/datasets/orvile/axial-ct-imaging-dataset-kidney-stone-detection.

The same framework, consisting of DINO self-supervised pretraining followed by DeiT-Tiny fine-tuning, was applied to this dataset and achieved a test accuracy of 97.03%, precision of 97.22%, recall of 96.88%, and F1-score of 97.01%. These results demonstrate consistent and balanced performance across both Stone and Non-Stone classes, supporting the generalizability of the proposed methodology on an independent dataset.

### 4.8 Clinical significance

The proposed framework may serve as a clinical decision support tool for the classification of kidney CT images into cyst, stone, tumor, and normal categories. By automatically identifying abnormalities, it may help streamline the image review process and support radiologists in clinical practice. The system is not intended to replace radiologist expertise but to provide supplementary information that may assist clinical interpretation and decision-making.

The best-performing architecture, DeiT-Tiny, achieved an inference time of 6.93 ms per image on a Tesla T4 GPU, allowing approximately 144 images to be processed per second. This indicates that the model is fast enough for real-time use in clinical settings with GPU hardware.

In addition to classification, the proposed framework includes explainability features based on Grad-CAM++ and DINO self-attention visualizations, which highlight the image regions that contributed most to each prediction. The framework also incorporates calibration analysis (Expected Calibration Error) to assess the reliability of model confidence scores. These features may help radiologists better understand the model’s predictions and support its use as a decision-support tool rather than a black-box system.

### 4.9 Practical and methodological contributions

Beyond classification accuracy, this study provides several additional contributions as summarized below.

*Self-supervised pretraining*: DINO self-supervised pretraining was employed to learn useful image representations from unlabeled CT scans before supervised fine-tuning. This reduces reliance on large, annotated datasets that are often difficult to obtain in medical imaging.

*Lightweight architecture*: The best-performing model, DeiT-Tiny, achieved 99.16% accuracy using only 5.6 million parameters and 34.3 million MACs. Compared with larger models such as ViT-Base (86.0 million parameters), DeiT-Tiny maintains strong performance while requiring fewer computational resources.

*Model Calibration*: To assess model reliability, ECE and reliability diagrams were used to indicate whether the model’s confidence scores accurately reflect the likelihood of correct predictions, supporting more reliable decision-making.

*Quantitative explainability*: Model interpretability was assessed using Grad-CAM++ sparsity and sensitivity scores and DINO attention diversity, entropy, concentration, and confidence metrics to provide insights into how the model makes its predictions.

Overall, the framework combines high accuracy, computational efficiency, interpretability, and reliable confidence estimates for kidney CT classification.

## 5 Conclusion

This study presents a novel two-stage deep learning framework for automated classification of kidney CT images into four categories: Cyst, Normal, Stone, and Tumor, using a CT-scan dataset of 12,446 images. In the first stage, a self-supervised learning approach based on DINO with a DeiT-Tiny backbone is utilized, where a student-teacher distillation mechanism facilitates the model to learn meaningful and discriminative feature representations without relying on labeled data. The pre-trained backbone was then fine-tuned for supervised classification using a custom MLP head. The proposed model achieved a test accuracy of 99.16% and was further validated using 5-fold stratified cross-validation, obtaining a mean validation accuracy of 98.41%, along with bootstrap-based 95% confidence intervals (0.9803 and 0.9875), showing that the model performs reliably and consistently across different data splits. In addition, model calibration was assessed using Expectation Calibration Error (0.0058), demonstrating that the predicted confidence scores are well-aligned and suitable for clinical interpretation.

To address interpretability in medical AI, two explainability methods were employed. Grad-CAM++ highlights important regions in the images and was evaluated using sparsity and sensitivity metrics with mean values of 0.782 and 0.0218, respectively, over 15 representative samples. In parallel, DINO’s multi-head self-attention was analyzed to capture diverse and clinically relevant spatial patterns across attention heads, assessed using metrics such as diversity, entropy, confidence, and concentration with mean values of 0.9317, 0.3332, 0.5661, and 0.8956, respectively, over 15 representative samples, to provide clear and measurable insights into the model’s decision-making beyond simple visual observation. The framework also demonstrated good generalizability, achieving 97.03% accuracy on an independent CT dataset from Iraq and maintaining robust performance from different sources.

## 6 Limitations and future directions

The CT-Kidney dataset does not include patient identifiers, as the information was removed before public release. As a result, the number of unique patients is unknown, and the dataset was split at the image level rather than the patient level. Therefore, it cannot be confirmed whether images are from the same patient and appear in both training and test sets. Future studies should use datasets with patient identifiers to enable patient-wise splitting and more rigorous evaluation.

This study was conducted primarily on a single public dataset and has not been externally validated in a real clinical setting. Although the proposed approach showed consistent performance when retrained on an independent public dataset, further validation using the same trained model on external clinical datasets is needed to confirm its generalizability.

The proposed framework was not compared with radiologist performance because expert reader annotations and a formal reader study were not available. Therefore, although the model achieved strong classification performance on the test set, its performance related to radiologists remains unknown. Further studies should compare the model with radiologists to better assess its clinical usefulness.

Future work may explore extending this framework to multi-modal imaging modalities, along with real-world clinical validation studies to examine its diagnostic effectiveness.

## Data Availability

The original contributions presented in the study are included in the article/supplementary material, further inquiries can be directed to the corresponding author.

## Footnotes

1
https://www.kaggle.com/datasets/nazmul0087/ct-kidney-dataset-normal-cyst-tumor-and-stone
